# Implantable Shock Absorber: Breakthrough or Hype?

**DOI:** 10.1007/s12178-026-10022-1

**Published:** 2026-03-25

**Authors:** Jared A. Nowell, David C. Flanigan

**Affiliations:** Department of Orthopaedic Surgery, The Ohio State University Wexner Medical Center, 2835 Fred Taylor Drive, Columbus, OH 43202 USA

**Keywords:** Implantable shock absorber, Knee osteoarthritis, Knee preservation, Medial knee pain

## Abstract

**Purpose of Review:**

The goal of this review is to discuss the current understanding of implantable shock absorbers (ISA) including mechanism of action, usage in patients, patient outcomes and the future of this technology.

**Recent Findings:**

Since the introduction of the ISA, it mainly has functioned as a surgical option for individuals with symptomatic medial compartment osteoarthritis who are too young, not indicated, or do not wish to proceed with arthroplasty. Biomechanically, ISA reduces peak medial compartment force by 32%. In a Food and Drug Administration (FDA) study, ISA was found superior to HTO, with significant reduction of pain and improvement of function. Survivorship and freedom to conversion to arthroplasty remains 85% at 5 years. Current randomized trial focuses on impact of continued non operative treatment of OA verses ISA.

**Summary:**

ISA is a reasonable surgical option for the treatment of medial compartment osteoarthritis without the need for disruption of the patient’s native anatomy through osteotomy or arthroplasty.

## Introduction

Osteoarthritis (OA) is a condition that affects an estimated 7.6% of the global population. This equates to roughly 595 million individuals who have OA affecting at least one joint. The knee is the most common joint comprising from 34.6% to 66.2% of all OA depending on the region of the world [1]. When looking further at knee OA it has been shown that roughly 50% of all knee OA is unicompartmental with only 17% of it involving all three compartments [2]. Over the next 25 years the impact of OA is expected to grow with an increase of 74.9% by the year 2050 [1]. 

Historically the initial treatment for knee OA has been a combination of non-operative modalities which included weight loss, physical therapy, activity modification, bracing and pharmacological interventions (nonsteroidal autoinflammatory drugs (NSAIDs), acetaminophen, tramadol, cyclooxygenase-2 inhibitors, intra-articular corticosteroids and hyaluronic acid as well as platelet rich plasma (PRP) [3, 4]. When these modalities no longer improve symptoms one may consider surgical interventions. Tricompartmental OA typically requires total knee arthroplasty (TKA) while unicompartmental knee OA may be treated with unicompartmental knee arthroplasty (UKA) or osteotomy in select patients [4–6]. Projections have shown that the volume of these procedures, specifically TKA, are only expected to increase through the year 2060 [7]. While arthroplasty procedures are effective they tend to be reserved for an older population given the limited longevity of implants and they require modification of a patient’s native anatomy. In the case of osteotomy, this requires an adjustment of alignment. Unfortunately, not all patients are candidates for arthroplasty due to age and activity level and they may prefer not to move to arthroplasty. More recently there has been the introduction of the implantable shock absorber (ISA) which provides treatment of medial compartment knee OA that does not require changes in patient’s native anatomy and is an extra-articular procedure.

## What is an Implantable Shock Absorber?

### Current Description

The ISA was first described by Clifford et al. in 2013, consisting of a distal femoral and proximal tibial base plate connected by a cobalt chromium alloy absorber [8]. All of the components were placed extra-articular and were stated to absorb a maximum load of 29 pounds during knee extension with the goal of offloading an arthritic medial knee compartment [8]. The ISA’s original iteration was the KineSpring Knee Implant System with a next generation implant known as the Atlas System being produced and illustrated in a case report by Slynarski et al. in 2017 for treatment of medial knee osteoarthritis in an ex-professional basketball player [9]. The most recent iteration of the ISA, medial implantable shock absorber or MISHA, obtained approval by the FDA in 2023 [10]. The MISHA consists of a 0.8 × 5 cm cylindrical polycarbonate urethane shock absorber fixed by low profile 2.5 × 4.0 cm titanium plates to the distal femur and proximal tibia. The implant is placed extra-articular and superficial to the medial collateral ligament (MCL) [10]. 

### Mechanism of Action

The ISA produces its offloading function through the absorber that is located in between the two base plates that are attached to the femur and the tibia. During stance the absorber engages and gradually compresses to help unload the medial knee compartment. As the knee transitions into the swing phase the absorber and the compressing piston disengage allowing this portion to elongate ensuring a smooth gait [11]. Overall, throughout a variety of studies the ISA has consistently shown the ability to offload the medial compartment of the knee.

Cadaver and clinical trials have investigated the results of the ISA to quantify the direct effect it has on values such as joint space width, contact pressure, and compartment pressure.

Miller et al. in 2015 studied 9 patients implanted with the ISA and followed them for 2 years [12]. At the end of their study they found the joint space width in the medial compartment of the treated knee increased from 0.9 mm at baseline to 3.1 mm which was statistically significant. Also, using fractal signature analysis (FSA) they demonstrated a decrease of 2.8% of the fractal signature of the vertically oriented trabeculae in the medial compartment which implies improved subchondral bone trabecular integrity.

Another study directly compared the unloading effect of ISA to that of HTO. By using cadaver knees and the KineSpring system Bode et al. demonstrated that the unloading effect of the ISA is similar to that of an HTO with both a 5 or 10 degree correction [13]. Using cadavers Kloos et al. showed that the ISA decreased medial contact pressure by 0.20 MPa compared to a native test cycle [14]. 

Lastly Morgan et al. used a validated three dimensional computer model of the tibiofemoral joint integrated in a computer assisted design of an ISA [11]. With their calculations they found that the ISA resulted in a mean decrease in medial compartment pressure of 188 N along with a decrease in the compressive force of 53%. This decrease in medial compartment translated to a peak force reduction of 32% as demonstrated by cartilage-meniscus stresses.

## Current Usages

### Medial Compartment Osteoarthritis

The primary indication of the ISA is for the treatment of symptomatic medial compartment osteoarthritis of the knee. Since the implant was first discussed in 2013 it has been used in case reports, prospective cohort studies and retrospective case control studies all for the treatment of medial compartment knee OA [9, 15–18]. The primary outcomes in these studies have all varied including patient reported outcome measures (PROMs), ability to return to sport, survivability to a secondary procedure and a comparison to high tibial osteotomy. Given the original rationale for creating the ISA, it is not surprising that the vast majority of literature focuses on this pathology.

### Medial Meniscus Root Repair

While the ISA has mainly been studied looking at medial knee OA, the effects it has on decreasing peak force on the meniscus open up opportunities for other usages. In a published case report Neijna used ISA in combination with a medial meniscus root repair to allow immediate partial weightbearing on the operative extremity for 2 weeks followed by full weight bearing as tolerated, instead of their typical 6 week non-weight bearing protocol [19]. They believe that the medial compartment off loading effects of the ISA help to protect the repair and allow quicker patient recovery as well as decreasing the difficulty with making patients non-weight bearing for 6 weeks. The surgical technique published does not provide patient outcomes, rate of retear or reinjury of the medial meniscus. Thus, this would be considered an off-label use and requires controlled studies to determine whether this could be indicated on a routine basis. However, it highlights how the ISA could potentially be used in conjunction with treatment of medial meniscus pathology to decrease periods of reduced weight bearing or contact pressure on the repairs.

### Indications and Contraindications

The ISA indications and contraindications have mostly stayed consistent while the technology has evolved. In the initial literature discussing the implant the manufacturer listed a single indication for its use of treatment of symptoms of pain and decreased function secondary to OA of the medial compartment of the knee [8]. In comparison there were 10 contraindications listed for the device. Contraindications were (1) active, local infection or previous intra-articular infection; (2) neuropathic or Charcot joint; (3) rheumatoid arthritis of the knee; (4) joint instability in the affected knee; (5) moderate-to-severe osteoporosis; (6) symptomatic lateral or patellofemoral OA in the affected knee; (7) varus alignment greater than 10° in the affected knee; (8) hyperextension greater than 10°; (9) severe deformity leading to impaired fixation or improper positioning of the implant; and (10) metal allergy or hypersensitivity [8]. Age, sex and obesity were not considered contraindications with the initial Kinespring device.

In the FDA Calypso Trial, the indications were patients aged 25–65 years with isolated medial compartment knee OA with Kellgren-Lawrence grade I-IV changes(KL) that have failed 6 months of nonoperative management [20]. Patients with a weight over 300 lbs and BMI > 35 kg/m^2^ were excluded in the trial. Contraindications include significant medial meniscus extrusion, significant medial joint line osteophytes, hip knee ankle angle of less than 15˚ of varus or flexion contracture > 10˚.

Since FDA approval, MISHA is indicated for symptomatic medial compartment OA (KL1-4). Patients may have asymptomatic arthritis in other compartments and still be appropriate for MISHA. Appropriate patients, regardless of age and BMI can be considered for MISHA. The contraindications of significant medial meniscus extrusion, large medial osteophytes, significant varus > 10˚, and flexion contracture remain as they can impact the implant function. Weight and BMI are relative contraindications as the device will still function but the amount of functional unloading will decrease with higher weight. It is also important to consider patients with a thin medial knee soft tissue envelope as this may increase the risk of implant prominence or wound complication.

## Patient Outcomes

### PROMs

The ISA has shown clinically meaningful improvement in patient’s pain and function across studies throughout each generation of the device. Miller in 2015 followed 9 patients implanted with the first generation ISA for 2 years. The primary outcome in this study was the Western Ontario and McMaster Universities Osteoarthritis Index(WOMAC) and found there was an improvement of 92% for pain, 91% for function and 79% for stiffness that was seen at two years [12]. This effect has been replicated by multiple other studies. Slynarski in 2019 followed 25 subjects for 2 years after the implantation of the second generation ISA system [18]. For this study the primary outcome was a clinically meaningful improvement in the WOMAC score defined as a ≥ 20% and ≥ 10 point absolute improvement. It was demonstrated that 100% of patients met the primary outcome for the WOMAC pain score and all but one patient achieved it for the WOMAC function score at two years. Additionally, only one patient had removal of the implant due to continued knee pain and stiffness.

Diduch et al. had the same criteria for WOMAC when identifying patients who were “responders” to the treatment of the ISA. In a prospective cohort study comparing ISA to HTO they had 81 patients who had the ISA and 81 patients with HTO and showed at 2 years that 95.8% of patients who were responders for WOMAC pain and 91.7% were responders for WOMAC function [10]. The Knee Injury Osteoarthritis Outcome score (KOOS) was investigated by Sherman et al. in order to understand the return to physical activity and sport for patients with an ISA. At 2 years they found a statistically significant increase of the KOOS from a mean score of 13.8 at baseline to 62.3 [16]. 

Looking across the ISA experience, Gomoll in 2023 showed similar positive effects on the WOMAC score with a minimum of 2 years of follow up with a maximum length of 5 years. This study followed 171 patients demonstrating that the mean WOMAC pain score decreased 71% from baseline from 58 to 16 points while the WOMAC function improved 69% decreasing from 56 to 17 points. The changes in the WOMAC score were both found to be statistically significant.

### Return to Activity and Sport

The literature on the ability of patients to return to sport or physical activity after ISA implantation is currently sparse. One of the first mentions of this was in a case report of an ex-professional basketball player in 2017. In Slynarski’s publication the follow up period was limited to 6 months but the patient had been able to return to recreational basketball at this time without any noted difficulties or limitations [9]. However the continued ability of competing is unknown as it is a single case report and no further follow up has been published.

**Fig. 1:**
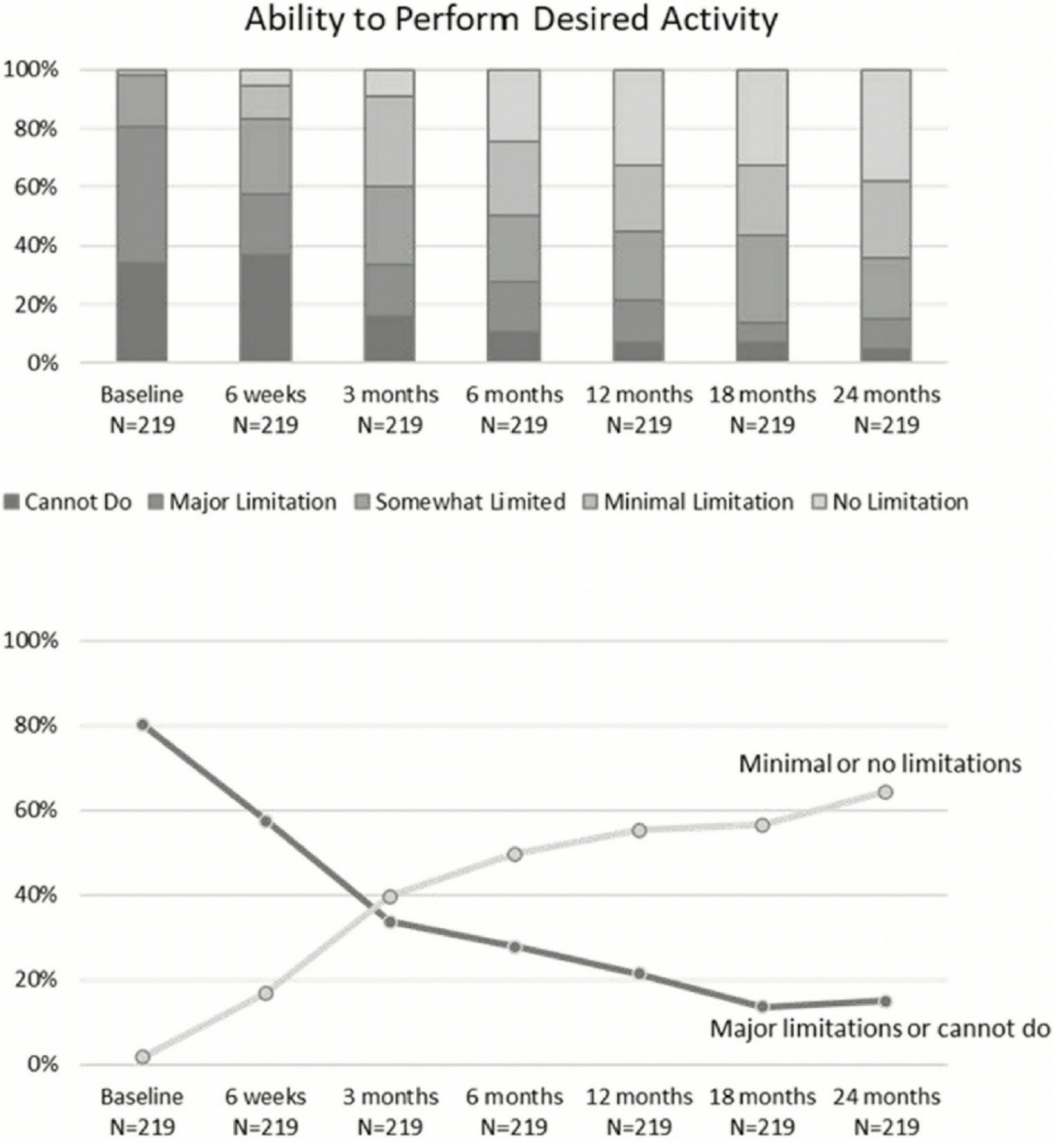
Top figure illustrates the functional limitations of activities for individuals over time before and after placement of implantable shock absorber. Bottom figure shows the proportion of activities with major limitations or cannot do decreased over time while proportion of activities with minimal or no limitations increased over time. Figure previously published in Journal of Cartilage and Joint Preservation by Sherman et al. in 2025 [16].

ISA has shown a positive impact on the ability to return to a variety of activities. Most recently Sherman in 2025 evaluated 73 patients for 2 years to establish their return to activities. At the beginning of the study patients self identified three activities in their lives that were limited by their osteoarthritis which encompassed 8 categories; running, leisure walking, non-contact and contact sports, cycling, moderate recreational activity, aerobics and gym workout. The categories of running and cycling both separately composed about 20% of all responses. In the beginning of the study 1.8% of activities could be performed with minimal or no limitation which improved to 64% by 2 years (Fig. 1). Further, 69% stated their knee never felt normal at baseline which reduced to 19% by the end of the study.

### Comparison to High Tibial Osteotomy

As the goal of the ISA is to reduce medial compartment pressure it is often compared to HTO which similarly has a goal of offloading the medial compartment. This was investigated directly by Diduch in conjunction with the FDA [10]. The study was a prospective cohort study comparing 81 patients with ISA propensity matched to 81 HTO patients collected in a previous study by Moximed, the ISA manufacturer. The primary endpoint consisted of the proportion of patients who met 5 criteria by the end of 2 years. The 5 criteria were (1) responder for WOMAC pain; (2) responder for WOMAC function; (3) freedom from deep infection requiring surgical intervention, damage to adjacent neurovascular or ligament structures necessitating reconstruction, or HTO nonunion; (4) maintained integrity of implant or hardware; (5) no conversion to arthroplasty or other joint modifying surgery. Responders were those with at least 20% improvement from baseline and an absolute change of greater or equal to 10 points. Implant removal was not a study failure for either arm [10]. ISA demonstrated superiority as 85.6% of ISA met the primary endpoint compared to 65.5% of HTO (Table 1). The ISA group also outperformed HTO in all five secondary endpoints that were outlined in the beginning of the study. It took the ISA group 13.4 days to achieve full weight bearing status in comparison to 58 days for the HTO group. Following the results of this study the FDA then approved the use of the 3rd generation ISA known as the MISHA in the United States.

**Table 1:** Five parameters of the primary endpoint for responders in the Diduch study for both the implantable shock absorber(ISA) and high tibial osteotomy(HTO group). Table previously published in Cartilage by Diduch et al. in 2023 [10]

	ISA Arm			HTO Arm		
Variable	*N*	*n*	%	*N*	*n*	%
Enrolled subjects	81	81	100	81	81	100
WOMAC pain responder	72	69	95.8	58	51	87.9
WOMAC function responder	72	66	91.7	64	52	81.3
No subsequent surgical intervention	81	80	98.8	81	80	98.8
No device-related SAE	81	77	95.1	81	76	93.8
Maintenance of implant or hardware integrity	81	80	98.8	81	80	98.8
Overall composite clinical success	81	–	85.6	81	–	65.5

*ISA* implantable shock absorber, *HTO* high tibial osteotomy, *WOMAC* Western Ontario and McMaster Universities Arthritis Index, *SAE* serious adverse event.

In 2025 Golant performed a review to summarize the current knowledge known about the ISA [21]. Part of the report noted that the ISA had a statistically significant lower revision rate of 13.6% compared to 75.3% for HTO. With the combined information for these two articles the ISA so far has outperformed HTO in the treatment of medial compartment knee OA.

### Implant Impact on Conversion to Arthroplasty and Survivability

The impact ISA has as a treatment modality for medial compartment knee OA to delay or eliminate the need for joint arthroplasty has been established. Pareek et al. performed a retrospective case control study of patients with medial knee OA and subchondral insufficiency fractures of the knee (SIFK) treated either with ISA or nonoperative care [15]. The study consisted of 42 total patients with 21 in each group. It demonstrated the one and two year freedom from arthroplasty was 100% in the ISA group compared to 76% and 55% respectively in the control group. The study further stratified outcomes using the SIFK score. When comparing low, medium and high risk SIFK score no difference between groups was noted for low SIFK score but it was statistically significant for the high risk group. None of the nonoperative care patients with a low SIFK score went on to arthroplasty but in the high risk group the survival rate was 33% and 0% at one and two years respectively. ISA was most strongly associated with avoidance of arthroplasty at two years in those with high risk SIFK scores. A subsequent study compared in a 2:1 match patients with SIFK, considered the control group and patients implanted with the ISA [22]. A total of 19 patients had the ISA compared to 38 controls. Survival rate from arthroplasty at 2 years was 100% in the ISA group compared to 61% in the control group. One patient in the ISA group did have hardware removal at one year.

**Fig. 2:**
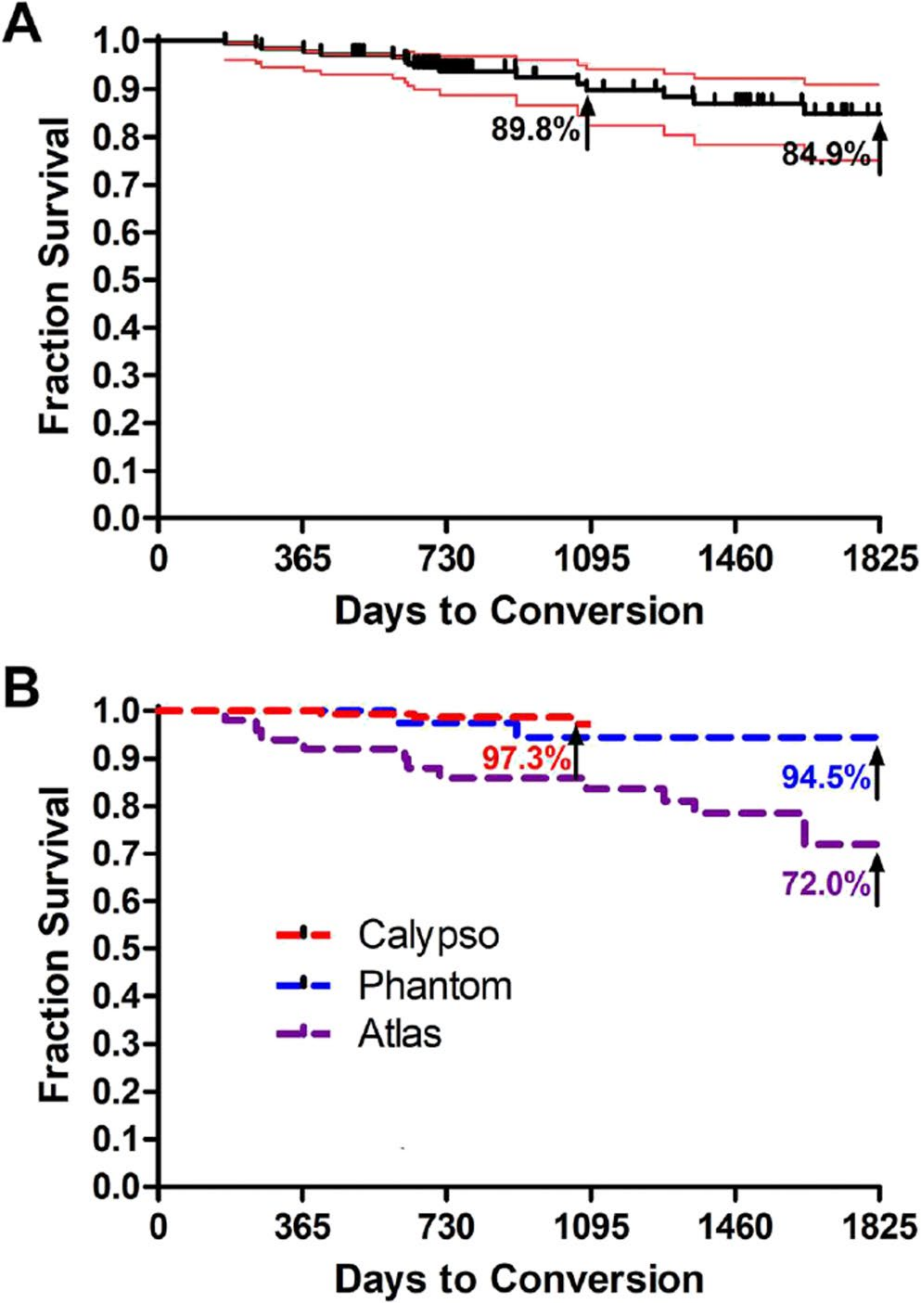
Top graph shows the overall rate of survival from conversion from implantable shock absorber to arthroplasty over time. Bottom graph shows the rate of survival from conversion by study with the Calypso being the most recent having the shortest follow up time. Figure previously published in knee surgery, sports traumatology, arthroscopy by Gomoll et al. in 2023 [23]

Further evidence of ability to avoid arthroplasty using ISA was expanded on by Gomoll et al. [23]. This study consisted of 171 patients with ISA spread across three different prospective single arm clinical trials that occurred during different time periods from 2014 to 2020. All patients were followed for a minimum of 2 years with a maximum length of 5 years with a primary outcome of survival without arthroplasty or HTO. At a mean of 3.2 years of follow up the overall survival rate was 90.6%. Using Kaplan-Meyer analysis the median 3 and 5 year survival from arthroplasty were 89.8% and 84.9% respectively. In addition, given the results were obtained from three separate prospective trials that occurred at different times the ISA design was modified and improved during data collection. Looking at each study independently it was found in the latest study, the Calypso study, which consisted of 81 patients, that the median 3 year survival rate was 97.3% (Fig. 2). Overall, this study aided in providing evidence of the efficacy of ISA as a surgical option for patients with symptomatic medial knee OA wanting to avoid arthroplasty or HTO.

### Complications

Sylnarski et al. found that the most common reported complication after ISA was knee pain seen in 15.4% of their 26 subjects (second generation ISA). One patient had the implant removed due to knee pain and stiffness after one year and no infections or deep vein thrombosis were noted [18]. In Diduch’s study the third generation ISA noted a total complication rate of 16.0% [10] The most common complications were pain at 4.9% and deep incisional surgical site infection both occurring at a rate of 4.9%. Other complications reported included discomfort(described as inability to perform certain tasks or as a catching or pulling sensation), hospital readmission and scar formation (Table 2). Diduch’s study included a comparison of the complication rates of HTO and ISA and the HTO group was found to have a statistically significant higher complication rate of 45.7% (*p* < 0.001). Pain was the most common in the HTO group at a rate of 45.7% which was statistically higher to the rate in the ISA group(4.9%). Deep incisional surgical site infection rate in the HTO group was 2.5% and was not found to be different from that in the ISA group(4.9%).

**Table 2:** All complications noted after placement of implantable shock absorber in comparison to those seen after high tibial osteotomy. Table previously published in Cartilage by Diduch et al. in 2023 [10]

	ISA Arm (*N* = 81)			HTO Arm (*N* = 81)			*P* value
	Events	Subjects	Rate %	Events	Subjects	Rate %	
Bleeding/Hematoma	0	0	0.0	1	1	1.2	1.000
Discomfort—catching or pulling sensations	1	1	1.2	0	0	0.0	1.000
Discomfort—inability to perform certain tasks, such as lifting and exercising	2	2	2.5	0	0	0.0	0.497
Hospital readmission-removal of implant ddue to dissatisfaction	1	1	1.2	0	0	0.0	1.000
Infection—deep incisional surgical site	4	4	4.9	2	2	2.5	0.682
Infection—superficial incisional site		0	0.0	1	1	1.2	1.000
Nerve injury (neuropathy)—injury to a nerve resulting in motor or sensory symptoms such as temporary or permanent weakness	0	0	0.0	1	1	1.2	1.000
Other	0	0	0.0	3	3	3.7	0.245
Pain	4	4	4.9	30	29	35.8	<.001
Psychological event	1	1	1.2	0	0	0.0	1.000
Scar formation—Other	1	1	1.2	1	1	1.2	1.000
Scar formation—periprosthetic adhesions/fibrosis	1	1	1.2	0	0	0.0	1.000
Swelling	0	0	0.	2	2	2.5	0.97
Wound—Other	0	0	0.0	1	1	1.2	1.000
All device- and procedure-related SAEs	15	13	16.0	42	37	45.7	<0.001

*ISA* implantable shock absorber, *HTO* high tibial osteotomy.

## Cost Effectiveness

The cost effectiveness relative to other surgical treatments has been evaluated. Bhandari et al. looked at the possible impact of the first generation ISA, the KineSpring, in comparison to conservative treatment, UKA, TKA and HTO using estimated cost models and assuming treatment durability of 10 years. In four separate studies in Spain, Italy, United Kingdom and Germany, Bhandari evaluated the calculated cost-utility ratio of the first generation model ISA and found the ISA does appear to be a cost effective treatment for medial compartment knee OA [24–27]. Utilizing a similar model to evaluate the results in Canada Bhandari demonstrated that the first generation ISA had a greater initial cost in surgery but the 10 year overall expected cost was $12,599 compared to $17,570 for UKA or $22,825 for HTO indicating in the long term the ISA may be a more cost effective treatment [28]. 

## Conclusion

The ISA was developed as an alternative option for treatment of symptomatic medial compartment knee OA to allow patients to continue their activities with limited alterations in comparison to other surgical options such as arthroplasty or HTO. While it is effective for medial compartment knee OA, ISA to be used as an unloading device with concomitant procedures need further exploration. Additionally, research is needed to understand the long term results of the ISA as well as the cost effectiveness of the more recent generations of the device.

### Human and Animal Rights

All reported studies/experiments with human or animal subjects performed by the authors have been previously published and complied with all applicable ethical standards (including the Helsinki declaration and its amendments, institutional/national research committee standards, and international/national/institutional guidelines).

## Key References

- Clifford AG, Gabriel SM, O’Connell M, Lowe D, Miller LE, Block JE. The KineSpring((R)) Knee Implant System: an implantable joint-unloading prosthesis for treatment of medial knee osteoarthritis. Med Devices (Auckl). 2013;6:69–76. doi: 10.2147/MDER.S44385.
  - ○ First literature published on implantable shock absorber explaining its rationale for use, design as well as the initial indications and contraindications.
- Diduch DR, Crawford DC, Ranawat AS, Victor J, Flanigan DC. Implantable Shock Absorber Provides Superior Pain Relief and Functional Improvement Compared With High Tibial Osteotomy in Patients with Mild-to-Moderate Medial Knee Osteoarthritis: A 2-Year Report. Cartilage. 2023;14(2):152 − 63. doi: 10.1177/19476035231157335.
  - ○ Direct head to head comparison of the implantable shock absorber to high tibial osteotomy demonstrating the noninferiority and superiority of the implantable shock absorber. Study performed in conjunction with the FDA allowing approval of the third generation of the implantable shock absorber.
- Gomoll AH, Diduch DR, Flanigan DC, Ranawat AS, Slynarski K, Walawski J, et al. An implantable shock absorber yields an 85% survival-from-arthroplasty rate through 5 years in working-age patients with medial compartment knee osteoarthritis. Knee Surg Sports Traumatol Arthrosc. 2023;31(8):3307-15. doi: 10.1007/s00167-023-07373-4.
  - ○ Demonstrated the ability of implantable shock absorbers to help with survival from conversion to arthroplasty in patients with medial compartment knee osteoarthritis.
- Golant AM, Allison; Raji, Yazdan; Sherman, Seth. The implantable shock absorber for medial compartment unloading of the knee: a scoping review. Journal of Cartilage & Joint Preservation. 2025. doi: 10.1016/j.jcjp.2025.100252.
  - ○ Most recent systematic review evaluating the implantable shock absorber using currently available literature.

## Data Availability

No datasets were generated or analysed during the current study.
